# A Yeast/*Drosophila* Screen to Identify New Compounds Overcoming Frataxin Deficiency

**DOI:** 10.1155/2015/565140

**Published:** 2015-10-11

**Authors:** Alexandra Seguin, Véronique Monnier, Amandine Palandri, Frédéric Bihel, Michael Rera, Martine Schmitt, Jean-Michel Camadro, Hervé Tricoire, Emmanuel Lesuisse

**Affiliations:** ^1^“Mitochondries, Métaux et Stress Oxydant”, Institut Jacques Monod, UMR7592 CNRS-Université Paris Diderot, Sorbonne Paris Cité, 15 rue Hélène Brion, 75205 Paris Cedex 13, France; ^2^Unité de Biologie Fonctionnelle et Adaptative (BFA), UMR8251 CNRS-Université Paris Diderot, Sorbonne Paris Cité, 4 rue M. A. Lagroua Weill Halle, 75205 Paris Cedex 13, France; ^3^Laboratoire d'Innovation Thérapeutique, UMR7200 CNRS-Université de Strasbourg, Faculté de Pharmacie, 74 route du Rhin, BP 60024, 67401 Illkirch Cedex, France

## Abstract

Friedreich's ataxia (FA) is a rare neurodegenerative disease which is very debilitating for the patients who progressively lose their autonomy. The lack of efficient therapeutic treatment of the disease strongly argues for urgent need to search for new active compounds that may stop the progression of the disease or prevent the appearance of the symptoms when the genetic defect is diagnosed early enough. In the present study, we used a yeast strain with a deletion of the frataxin homologue gene as a model of FA cells in a primary screen of two chemical libraries, a fraction of the French National Chemical Library (5500 compounds) and the Prestwick collection (880 compounds). We ran a secondary screen on* Drosophila melanogaster* flies expressing reduced levels of frataxin during larval development. Half of the compounds selected in yeast appeared to be active in flies in this developmental paradigm, and one of the two compounds with highest activities in this assay partially rescued the heart dilatation phenotype resulting from heart specific depletion of frataxin. The unique complementarity of these two frataxin-deficient models, unicellular and multicellular, appears to be very efficient to select new compounds with improved selectivity, bringing significant perspectives towards improvements in FA therapy.

## 1. Introduction

Friedreich's ataxia (OMIM #229300, FA) is the most prevalent form of autosomal recessive spinocerebellar ataxia in Caucasians. It is a rather heterogeneous disorder characterized by progressive ataxia and dysarthria [1] usually appearing around puberty, but sometimes much later in life (>60 years). Neurological features include sensory neuropathy, deep sensory impairment, and signs of pyramidal-tract involvement. Nonneurological manifestations include hypertrophic cardiomyopathy (in ~60% of patients) and diabetes (in ~30% of patients). Friedreich ataxia is caused by mutations in the FXN gene, most frequently (96%) arising from an unstable hyperexpansion of GAA triplet repeat in the first intron of the gene [2] which results in decreased transcription of the FXN locus (and to some extent the adjacent PIP5K1B locus [3]) and reduced level of frataxin.

Frataxin is a highly conserved protein with homologues found in bacteria, yeast, invertebrates, plants, and mammals. In eukaryotic cells, the protein is synthesized with a presequence that targets the protein to the mitochondrial matrix. Most of our knowledge about the role of frataxin comes from studies of mutant yeast cells and cells from FA patients (reviewed in [4, 5]). The precise role of frataxin is still a matter of debate. It is generally recognized to participate in iron-sulfur cluster (ISC) assembly [6–9], but its function as an iron-chaperone in ISC synthesis remains fragile as illustrated by contradictory reports [10–14]. It was rather suggested that frataxin activates the transsulfuration reactions required for ISC biosynthesis [15], and a point mutation in the Fe-S scaffold protein Isu1p bypasses frataxin deletion in yeast [16]. Key features of frataxin-deficient yeast cells, FA patients fibroblasts, and virtually all models generated so far are a hypersensitivity to oxidative insult and the inability to induce proper antioxidant defenses [17–19]. In fibroblasts, this hypersensitivity to oxidative insult has been ascribed to impair signaling of antioxidant defenses resulting from actin stress fibers disorganization [19–21].

The therapeutic arsenal to treat FA is limited and several attempts aim at developing new strategies to better handle this fatal disease. The current trials focus on lowering oxidative insults (e.g., Idebenone, Phase III trial), reducing iron-mediated toxicity (e.g., deferiprone, Phase II trial), increasing antioxidant defense levels (e.g., pioglitazone, Phase III trial), or increasing frataxin expression (e.g., polyamides or erythropoietin or gene therapy) (for general reviews on therapeutic approach to FA, see [22, 23]).

Despite many efforts to overcome any of the abnormalities related to frataxin deficiency, there is currently no efficient treatment to cure or even stop the progression of the disease, mostly because many aspects of the pathological consequences of frataxin depletion are still not fully understood. As a result there is still a need to use new approaches and to identify new molecules to successfully fight FA. Unfortunately, the important genetic instability of frataxin knockdown cell lines, such as murine fibroblast models for Friedreich's ataxia, is a severe limitation in a high-throughput drug screen [24]. Cotticelli et al. [25] recently reported a high-throughput screening of several chemical libraries using a yeast strain with the frataxin gene (*YFH1*) under the control of a galactose inducible/glucose-repressible promoter to mimic frataxin deficiency. Based on a test to evaluate mitochondrial energetics, the screen allowed identifying a number of compounds that were further evaluated on the murine myoblast cell line C2C12 treated with ferric ammonium citrate and buthionine sulfoximine to recapitulate some of the phenotypes of FA cells. In the present study, we used a genuine* yfh1*-deleted yeast strain (Δ*yfh1*) as a model of FA cells in a primary screen of two chemical libraries, a fraction of the French National Chemical Library (5500 compounds, about 12% of the full chemical library) (http://chimiotheque-nationale.enscm.fr/) and the Prestwick collection (880 compounds) (http://www.prestwickchemical.com/). We used as a secondary screen genetically engineered* Drosophila melanogaster* flies expressing reduced levels of dfh that exhibit a strong developmental phenotype [26]. The complementarity of these two frataxin-deficient models, unicellular and multicellular, allowed the identification with an improved selectivity of 6 new compounds with high specific activity in both paradigms, one of them also active in improving heart functions in* Drosophila* with reduced frataxin expression in cardiomyocytes, bringing significant progress towards perspectives in FA therapy.

## 2. Materials and Methods

### 2.1. Yeast Strains and Growth Conditions

The* S. cerevisiae* strains used in this study were the cycloheximide resistant wild-type (WT) strain derived from YPH499 (MATa* ura3-52 lys2-801 ade2-101 trp1-*Δ*63 his3-*Δ*200 leu2-*Δ*1 cyh2*) and its derivative YPH499Δ*yfh1* (Δ*yfh1*::TRP1). To prevent the accumulation of extragenic suppressor mutations, the Δ*yfh1* mutant was constructed using the YPH499* yfh1* shuffle strain [27]. In the shuffle strain, the Δ*yfh1* deletion is covered by pRS318-*YFH1*, a plasmid containing the CEN,* CYH2,* and the* YFH1* HindIII genomic fragment. The plasmid was removed by counter selection in anaerobiosis on YPD-TE medium (1% yeast extract, 2% Bacto peptone, 2% glucose, 0.2% Tween 80, 20 mg·L^−1^ ergosterol) containing 10 *μ*g·mL^−1^ cycloheximide, which is toxic in the presence of the* CYH2* allele. To monitor the loss of mitochondrial DNA leading to a rho° status, the YPH499Δ*yfh1* strain was regularly crossed with an appropriate Rho tester strain and analyzed using standard yeast genetics procedures [28]. Only isolates with more than 90% Rho^+^ cells were used in the screening procedure. Control strains were the wild-type strain BY4741 (Mat a* his3-*Δ*1 leu2-*Δ*0 met15-*Δ*0 ura3-*Δ*0*) and its Δ*ggc1* derivative (Mat a* his3-*Δ*1 leu2-*Δ*0 met15-*Δ*0 ura3-*Δ*0 ggc1*::KANMX4).

### 2.2. Screening Procedure on Yeast

The chemical libraries were available as a series of 96-well microtiter plates containing the compounds as 0.01 M stock solutions in DMSO. Fresh isolates of YPH499Δ*yfh1*, obtained after plasmid shuffling, were maintained on YPD medium. To screen the chemical libraries, the cells were grown on minimum medium in which frataxin-deficient cells can only grow very poorly. This medium (YNB-Raf) consisted of Yeast Nitrogen Base (Difco) 6.7 g·L^−1^, supplemented with the required amino acids and 200 mg·L^−1^ adenine and 2% raffinose plus 0.1% glucose as carbon sources. 96-well microtiter plates containing 120 *μ*L YNB-Raf per well were inoculated at an initial OD_600 nm_ of 0.01. The chemicals were added at a final concentration of 10 *μ*M. The plates were incubated at 30°C for 3 days, and the cell density was measured by reading the optical density (OD_600 nm_) using a SpectraMax i3 microtiter plate reader (Molecular Device).

Dose-dependence of the compounds was tested (1) by monitoring the Δ*yfh1* cell growth on liquid cultures in YNB-Raf medium and also (2) by agar disc diffusion assays, as described in [29]. Two hundred forty microliters of exponentially growing cell cultures, adjusted to an OD_600 nm_ of 0.01, was mixed with 10 mL Ultrapure low melting point agarose (0.8% weight/vol in water, Invitrogen) maintained at 30°C after melting and poured onto a square Petri dish (12 cm × 12 cm) containing YNB-Raf solid medium. Sterile paper discs distributed on the agar plates with the lawn of Δ*yfh1* cells were impregnated with 7 *μ*L of each compound (10 mM in DMSO), and growth of the cells around the discs was monitored by scanning the plates at different time points.

### 2.3. Drosophila Stocks, Culture Methods, and Treatment with Compounds

UAS-fhRNAi (w[1]; Pw[+mC]=UAS-fh.IR2), UAS-mitoGFP (w[1118]; Pw[+mC]=UAS-mitoGFP.AP2/CyO), and da-GAL4 (P{GAL4-da.G32}2) were obtained from the Bloomington Stock Center. Hand-GS is described in [30]. Stock solutions of the tested compounds (10 mM in DMSO), or similar volumes of DMSO for control conditions, were incorporated in food medium (60 g·L^−1^ yeast, 34 g·L^−1^ corn meal, 50 g·L^−1^ sucrose, 8.6 g·L^−1^ agar, and 25 mL·L^−1^ methyl 4-hydroxybenzoate (200 g·L^−1^ in ethanol)) to a final concentration of 10 *μ*M or 50 *μ*M. To test the compounds on the defective pupariation, female da-GAL4 female flies were crossed with UAS-fhRNAi or w[1118] males and allowed to lay eggs for 3 hours on standard food medium at 26°C. 24 hours after egg laying, first instar larvae (L1) were collected and transferred at 23°C on food medium containing the tested compounds. Three to four samples of 50 L1 were transferred for each tested compound, and the timing of pupariation of these larvae at 23°C was followed up. This 23°C breeding temperature was chosen because, using our standard rearing medium, it led to a final percentage of pupariation of 50% for untreated frataxin-deficient larvae, a percentage well suited to identify both enhancement or suppression of the deleterious phenotype. Statistical significance of differences between treated and untreated larvae was assessed with one tailed *t*-test analysis. To test the compounds on the heart phenotype, expression of fhRNAi was driven by the heart specific RU486-inducible Geneswitch driver Hand-GS in UAS-mitoGFP;HandGS>UAS-fhRNAi flies as described in [31]. The activity of the Hand-GS driver (and hence the level of frataxin depletion) was controlled by RU486 added to the fly food (40 ng·mL^−1^ of food during development and 100 *μ*g·mL^−1^ during adulthood). The driver was simultaneously used to express a mitochondrial GFP, providing sufficient fluorescence in cardiomyocytes for high-speed video recording through the cuticle of anaesthetized flies.

### 2.4. *In Vivo* Imaging of Fly Hearts

UAS-mitoGFP;HandGS>UAS-fhRNAi and UAS-mitoGFP;HandGS>+ 4-day-old adult flies were anesthetized with Triethylamine (FlyNAP). Video movies were acquired on a Zeiss SteREO Lumar.V12 Stereomicroscope, with a NeoLumar S 1.5x objective as described in [30]. For every video, the 501 frames were flattened into one by using the ImageJ function Zproject (Max Intensity). The picture generated was thresholded for light intensity by using the set AutoThreshold function. The anterior part of the heart (abdominal segments A1/A2) was then detected with the Analyze Particles tool from ImageJ (minimum size = 6,000; maximum size = 100,000; circularity = 0–0.99). The vertical row used to measure the diastolic diameter was automatically positioned using the XM variable as the abscissa origin. Statistical significance was assessed by nonparametric Wilcoxon analysis.

## 3. Results

### 3.1. Screening of the Chemical Libraries on the Basis of Growth Rescue of the Δ*yfh1* Yeast Strain with Raffinose as the Main Carbon Source

**Figure 1:**
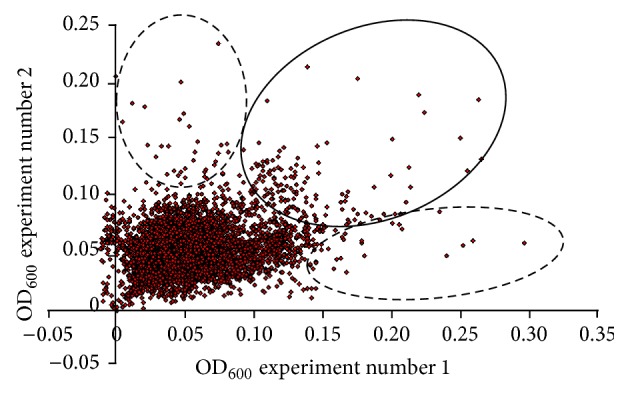
Drugs ability to improve the fitness of frataxin-deficient yeast cells. Pairwise analysis of the growth of the Δ*yfh1* cells in two independent experiments, using the full set of compounds from the French National Chemical Library (5500 compounds) and the Prestwick collection (880 compounds). The regions of the graph circled with dotted lines representing the results of growth improvement in only one condition are maybe therefore attributed to growth of extragenic suppressors. Compounds within the area circled with a solid line were typical of drugs entering the secondary screening in the yeast assay.

**Figure 2:**
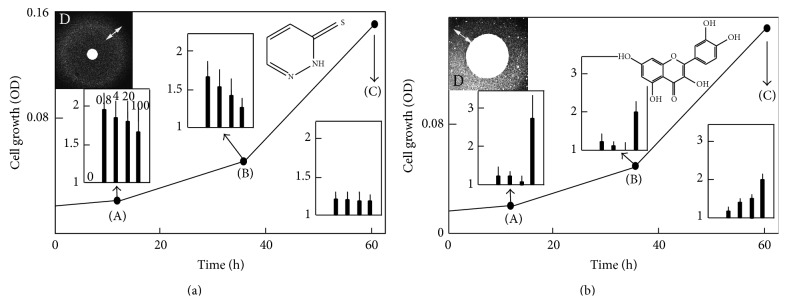
Effect of drugs on the kinetics of Δ*yfh1* cell growth. Panels A (effect of LPS-01-04-L-G10) and B (effect of LPGS-02-C06) show a typical growth curve of Δ*yfh1* cells in liquid YNB-Raf medium with no addition as a reference curve and in inserts, the effect of the drugs on the kinetics of growth evaluated by measuring the cell density (OD_600 nm_) at 3 different stages of the growth: 12 h (A), 36 h (B), and 60 h (C) after addition of the drug at different concentrations (0.8, 4, 20, and 100 *μ*M). The values in insert graphs represent the increase of growth due to the drug at these various concentrations, as *n*-fold increase of the cell density compared to the DMSO control (1 = no change, 2 = 2-fold increase, etc.). Dose-dependent effect is also illustrated by agar disc diffusion assays (YNB-Raf/agar medium) in panels D: paper discs (diameter of 0.3 cm) were impregnated with 7 *μ*L of the concentrated drugs (10 mM in DMSO), and the pattern of growth of Δ*yfh1* colonies around the discs was photographed after 2–4 days. The zones showing the highest density of colonies are indicated by a double arrow. All of the experiments were performed in quadruplicate.

**Figure 3:**
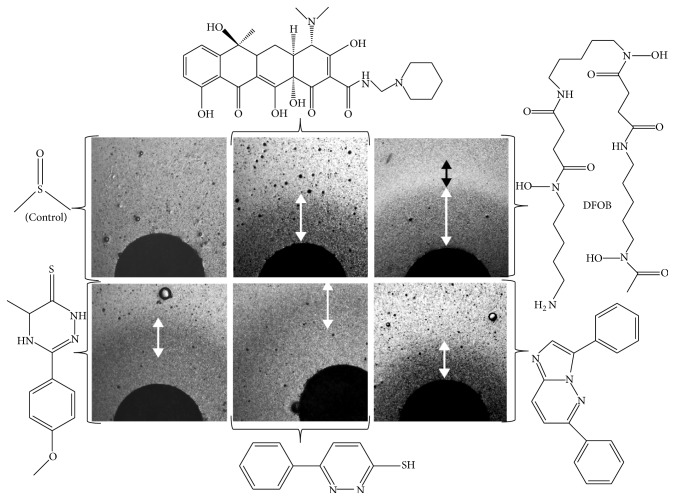
Dose-dependent effect of some drugs evaluated by agar diffusion assays. The agar diffusion disk assays were performed as described in Figure 2. White double arrows represent zones of growth improvement whereas black double arrows represent zones of growth inhibition. Pictures are shown in negative to highlight the contrast. Chemical used were from left to right upper panel DMSO (as a control), rolitetracycline (not evaluated further), and desferrioxamine B (DFOB) and lower panel LPS02-25-L-E10, LPS01-03-L-F03, and LPS02-30-L-H10.

Frataxin-deficient yeast cells (and more generally yeast mutants affected in oxidative phosphorylation) show a slow growth phenotype when raffinose is provided as the carbon source [32]. This is because raffinose, unlike glucose, prevents catabolic repression: full utilization of this carbon source thus requires the functioning of both the glycolytic and the oxidative phosphorylation pathways. We used this carbon source in our screen, rather than glycerol (which can only be metabolized by respiration) because we looked for drugs that would improve the mitochondrial functions without necessarily fully restoring them. A primary screening was run in triplicate on all the compounds, and results were compared pairwise. Typical results from one pairwise analysis out of three are presented in Figure 1. The regions of the graph circled with a dotted line represent conditions where the growth rescue is not consistent in the replicates and is most likely due to the appearance of genetic suppressors. The compounds from the region of the graph circled with a solid line were good candidates as active drugs. We selected the 60 compounds that were the most efficient at reproducibly improving the growth of Δ*yfh1* cells in our selection medium (YNB-Raf). In a validation screen, these compounds were tested on the growth of 3 yeast strains under the same experimental conditions: cells of the wild-type strain, Δ*yfh1* cells, and cells of a Δ*ggc1* strain, a strain which is also defective in iron-sulfur cluster biogenesis due to a lack of the mitochondrial GDP/GTP exchanger but has normal frataxin content [33]. This screen allowed us to select 18 molecules which improved the growth of Δ*yfh1* cells, but which had lower (or no) effect on the growth of Δ*ggc1* cells (data not shown). These molecules were studied in a wide range of concentrations (0.1–100 *μ*M) for their effect on the growth kinetics of Δ*yfh1* cells in liquid YNB-Raf medium. Dose-dependence of the compounds was also tested by agar disc diffusion assays [29]. The effects of the 18 selected compounds were very different according to their concentration and to the growth phase of yeast cells. Some examples are illustrated in Figures 2 and 3.  Figure 2 shows a typical growth curve of Δ*yfh1* cells in liquid YNB-Raf medium: there is a very long lag period (about 24 h) before the cells enter a short exponential phase of growth, and then the cells stop growing (stationary phase) to reach a maximum OD_600 nm_ value of about 0.2 (2 million cells·mL^−1^) after more than 3 days. We tested the effect of the selected compounds (added at different concentrations in the medium) on these different phases of growth: lag period, beginning of exponential phase, and end of exponential phase (A, B, and C, respectively, in Figure 2). We observed very different dose-dependent effects of the molecules on the growth of frataxin-deficient cells, allowing define categories of compounds acting at different concentrations on different phases of the growth. Examples are shown in Figure 2: some molecules strongly improved the early phases of growth of frataxin-deficient cells at low concentration (0.8 *μ*M) (LPS 01-04-L-G10, Figure 2(a)); some molecules improved all phases of growth at high concentration (100 *μ*M) (LPGS-02-C06, Figure 2(b)), while other molecules had different effects on the growth phases according to their concentration (data not shown). These differences in the effects of the selected compounds were also evidenced in the agar disc diffusion assays: maximum efficiency of a drug at low concentration resulted in a concentric zone of colonies growing better at some distance of the paper disc (Figure 2(a)), while colonies grew better in the immediate vicinity of the paper disc when the drug was more active at the highest concentration (Figure 2(b)). Variants of these patterns (zones of growth improvement/inhibition around the paper discs) were observed with different drugs (Figure 3). These results suggest that the different compounds selected in our screen improved Δ*yfh1* cells viability through distinct molecular mechanisms. One of these compounds was desferrioxamine B (DFOB) provided as deferoxamine mesylate (Figure 3). DFOB is the metal-free form of ferrioxamine B (FOB), the ferric iron complex of this siderophore for which* S. cerevisiae* has a specific transporter, Sit1p [34]. The beneficial effect of this strong iron chelator on Δ*yfh1* cell growth was proportional to its concentration (Figure 3). We also tested the effect of DFOB versus FOB: the iron-containing molecule was more efficient at rescuing cell growth than the iron-free one (data not shown).

**Figure 4:**
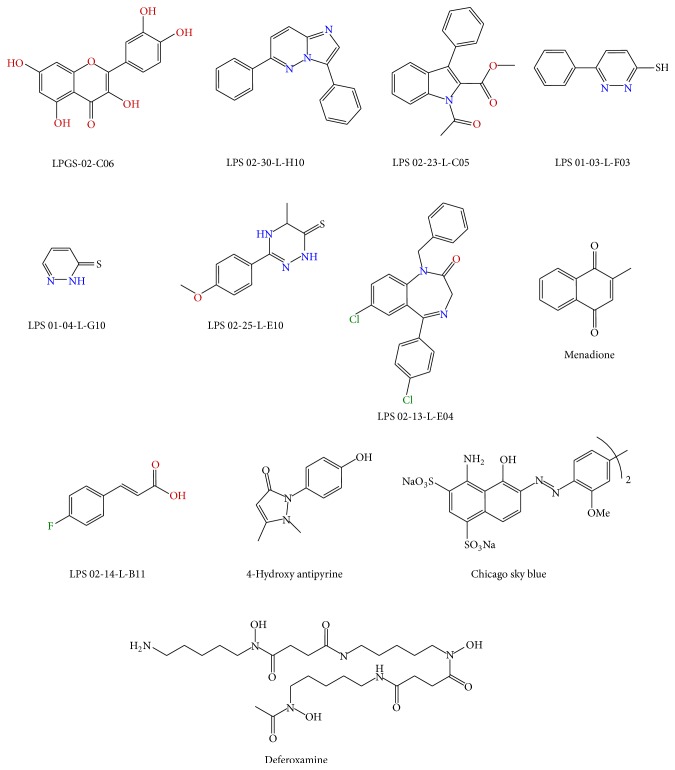
Chemical structure of the compounds selected from the yeast-based screen and used in the* Drosophila* based developmental assay.

Only the compounds presenting a strict specificity toward the Δ*yfh1* phenotypes and exhibiting no cytotoxicity (i.e., no growth inhibition at high concentration) were selected for evaluation of their efficiency* in vivo* in a* Drosophila* model of FA. The 12 selected compounds (8 from the “French National Chemical Library” and 4 from the Prestwick collection) are presented in Figure 4.

### 3.2. Evaluation of Drug Efficiency* In Vivo* on a* Drosophila* Model of FA

In* Drosophila*, several models have been developed to downregulate dfh (the ortholog of fxn) in various tissues by an UAS-GAL4 transgene based RNAi method [26, 31, 35–37]. Ubiquitous inactivation of dfh throughout development, under control of the ubiquitously expressed da-GAL4 driver, leads to a developmental blockage at the third larval stage. Frataxin-deficient larvae do not formed pupae at the expected time, continue to grow, and become giants. Only a fraction of these larvae undergo pupariation much later than controls. The frataxin-deficient larvae also present reduced activities of ISC-containing mitochondrial aconitase and of respiratory complexes II, III, and IV along with hypersensitivity to iron [26].

**Figure 5:**
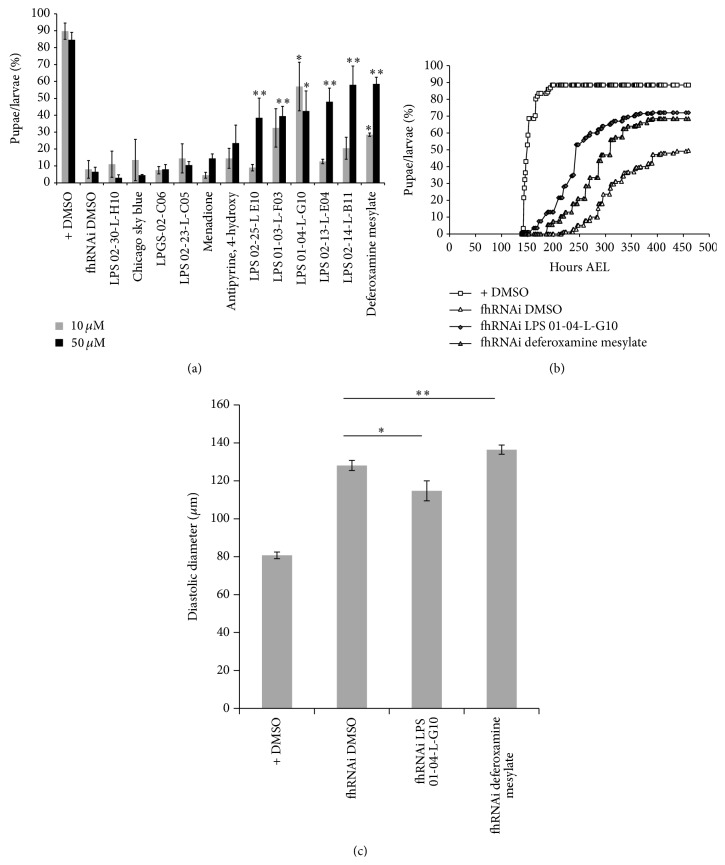
Drugs ability to rescue* in vivo* phenotypes induced by frataxin inactivation in* Drosophila*. (a) The timing of pupariation of da-GAL4>+ (+) and da-GAL4>UAS-fhRNAi (fhRNAi) larvae untreated (DMSO controls) or treated with compounds was followed. Percentages of larvae reaching pupariation 263 hours after egg laying (AEL) are shown. The 12 compounds selected in yeast were tested at 10 *μ*M (grey bars) and 50 *μ*M (black bars). Each treatment condition was tested on 3 to 4 samples of 50 larvae. All values are means (±SEM). Significant differences of da-GAL4>UAS-fhRNAi treated with a compound compared to untreated larvae of the same genotype are indicated: ^∗^ *P* < 5.10^−2^ and ^∗∗^ *P* < 5.10^−3^. (b) Percentages of larvae reaching pupariation as a function of time after egg laying for control larvae (+ DMSO) and frataxin depleted larvae and untreated (fhRNAi DMSO) and treated with 10 *μ*M deferoxamine mesylate or LPS 01-04-L-G10 are shown. (c) Diastolic diameters of 4-day-old UAS-mitoGFP;HandGS>+ control (*n* = 24) and UAS-mitoGFP;HandGS>UAS-fhRNAi adult male flies untreated (*n* = 26) or treated during development with 10 *μ*M LPS 01-04-L-G10 (*n* = 8) or deferoxamine mesylate (*n* = 17). All values are means (±SEM). Significant differences between treated and untreated UAS-mitoGFP;HandGS>UAS-fhRNAi flies are indicated: ^∗^ *P* < 5.10^−2^ and ^∗∗^ *P* < 5.10^−3^.

Therefore, we tested here the ability of the 12 compounds selected in yeast to rescue this developmental phenotype. To this purpose, we followed the timing of pupariation of da-GAL4>UAS-fhRNAi larvae treated with compounds at 10 or 50 *μ*M. In our breeding conditions, more than 80% of da-GAL4/+ control larvae formed pupae between 140 hours and 190 hours after egg laying (AEL) (Figure 5(b)). As expected, da-GAL4>UAS-fhRNAi larvae presented delayed pupariation: at 263 hours AEL, only 6–8% of these larvae have formed pupae (Figure 5(a)), and 50% never reached pupariation (Figure 5(b)). Two compounds, LPS 01-04-L-G10 and DFOB, improved both the timing of pupariation and the final percentage of larvae reaching pupariation when larvae were treated at 10 *μ*M (Figure 5(b)). Interestingly, the effect of LPS 01-04-L-G10 was more pronounced at the lower dose (10 *μ*M), as was observed in yeast (Figure 2(a)). The dose-dependent effect of DFOB was also similar to that found in yeast: 28.5% of the frataxin depleted larvae reached pupariation at 263 hours AEL with 10 *μ*M treatment and 58.5% with 50 *μ*M treatment (Figure 5(a)). FOB was not tested at this stage. At 50 *μ*M, we detected 4 additional compounds, LPS01-03-L-F03, LPS 02-14-L-B11, LPS02-13-L-E04, and LPS02-25-L E10, with significant improvement of pupariation (Figure 5(a)). Consequently, 50% of the compounds selected in yeast appeared to be active in flies.

Finally, we investigated whether the two most promising compounds (LPS 01-04-L-G10 and deferoxamine mesylate), active at the lower concentration (10 *μ*M), could have also beneficial effects on the heart dilatation phenotype induced by frataxin depletion in cardiomyocytes. Using the strategy recently described in [31], we measured diastolic diameters of 4-day-old UAS-mitoGFP;HandGS>UAS-fhRNAi adult male flies untreated or treated during development with 10 *μ*M of these compounds. As expected, untreated flies presented heart dilatations compared to age-matched UAS-mitoGFP;HandGS>+ control flies (Figure 5(c)). While treatment with deferoxamine mesylate significantly enhanced the pathological phenotype, we observed a significant rescue with LPS 01-04-L-G10 treatment (Figure 5(c)). Thus, this last compound rescues at least two pathological hallmarks of FA in* Drosophila* models and deserves attention as a leading compound for further improvements.

## 4. Discussion

Two chemical libraries were screened on frataxin-deficient yeast (*S. cerevisiae*). The first chemical library is the Prestwick collection (880 compounds) and is composed of approved drugs (FDA, EMA, and other agencies). This collection is particularly valuable as the compounds were selected for their known bioavailability and safety in humans. The second chemical library is a subset of the French National Chemical Library, composed of 5,500 compounds. This collection includes a large diversity of functionalized and drug-like compounds, mainly based on heterocyclic scaffolds. Indeed, this library is composed of chemical compounds synthesized by French medicinal chemists over the last decades. The screening was performed in triplicate at a single concentration (10 *μ*M) for each tested compound and led to identify 60 compounds that significantly improved the growth of frataxin-deficient yeast cells in liquid medium with raffinose as the main source of carbon. This set of compounds was clustered on the basis of structural features, dose-dependent and specific action on Δ*yfh1* cells, availability, and purity, in order to afford 12 molecules representative of the different chemical families. In order to assess the relevancy of each chemical family towards Friedreich's ataxia, the 12 selected compounds were tested on an animal model of FA, that is, the* Drosophila* model where frataxin-deficient larvae show impairments in the larval to pupal transition, a phenotype previously shown to be associated to decreased activities of several ISC containing enzymes [26]. The 12 compounds were tested for their ability to improve the defective pupariation due to frataxin ubiquitous inactivation. Four approved drugs were identified in yeast from the Prestwick collection (Menadione, Chicago Sky Blue, antipyrine, and desferrioxamine B), but only DFOB was confirmed to be active* in vivo* on the* Drosophila* pupariation assay. Known as an iron chelator, this drug is clinically used under its mesylate salt form to remove excess iron from the body. After 263 hours in presence of 50 *μ*M of DFOB, about 60% of frataxin-mutated larvae had undergone the pupariation step. Yeast cells have a specific transporter for ferric chelate FOB (Sit1p). Owing to the very high binding constant of ferric iron to DFOB (*K*_*D*_ 10^−31^ M, [38]), it is most likely that the DFOB provided to the cells will chelate the ferric iron present in the growth media and therefore be used by the cells as an iron source. Therefore, the rescuing effect of DFOB in the yeast model could be interpreted in different ways: either the drug acts as a classical iron chelator, by decreasing the total cell iron pool available to the cells, or it makes iron more available to the cells by preventing iron precipitation in the mitochondria. This latter hypothesis seems the more likely, since FOB also improved growth of frataxin-deficient cells. Moreover, iron chelators that cannot be used as iron sources by* S. cerevisiae* (bathophenanthroline disulfonic acid, ferrozine, 2, 2′-bipyridyl, salicyl-hydroxamic acid) and that are known to generate iron depletion in yeast did not rescue growth of Δ*yfh1* cells and had even toxic effects ([33] and data not shown). The rescuing effect of DFOB in the* Drosophila* model could be based on a different molecular mechanism than in yeast, although nothing is known about the possible use of FOB as an iron source by* Drosophila*. We are currently testing the effect of FOB versus DFOB in this model.

4-hydroxyantipyrine, one of the main metabolites of the nonsteroidal anti-inflammatory and antipyretic drug antipyrine, showed a slight effect, but this effect was not statistically significant. Surprisingly, menadione was found inactive in the developmental assay in flies. Also named vitamin K3, menadione shows both pro- and antioxidant activities [39] and was previously identified as a hit by Cotticelli et al. [25] in yeast depleted of Yfh1p. The mode of action of menadione is complex. Although the toxic effects of high doses (mM range) of menadione involve reactive oxygen species production [40] and depletion of intracellular glutathione pools [41, 42], pretreatment of yeast cells with low doses of menadione induces a protection against further oxidative insult [40]. The low dose used in our screen (10 *μ*M) may induce such a protective effect in the context of the Δ*yfh1* cells where exposure to oxygen induces both the production of reactive oxygen species and a depletion in intracellular glutathione pools (reviewed in [5]). Eight compounds from the French National Library were tested on the* Drosophila* pupariation assay, and 5 of them showed significant effects. The best compound is 4-fluorocinnamic acid (LPS 02-14-L-B11) which showed similar beneficial effect than deferoxamine in the pupariation assay. Interestingly, in another assay (rescue of the heart dilatation phenotype induced by heart specific frataxin depletion) deferoxamine mesylate increases the pathological phenotype, when LPS 01-04-L-G10 treatment significantly improves it. This points out that frataxin depletion likely impact several pathways whose relative importance may vary between tissues. Therefore one compound targeting one of these pathways may be active in one paradigm but inefficient to rescue another tissue-specific phenotype where a different pathway may be critical. LPS 01-04-L-G10, which is active in two assays in* Drosophila*, is a cinnamic derivative presenting a simple structure and is a good starting point for a hit-to-lead optimization process. In particular, the carboxylic acid function is expected to prevent the crossing of biological barriers and should be modified. Next, the 1,4-benzodiazepin-2-one (LPS 02-13-L-E04) was identified as an efficient compound at 50 *μ*M (about 50% of pupae). This result is promising as many benzodiazepines have been developed as anxiolytic drugs (ex: diazepam). Interestingly, this compound is a derivative of a TSPO ligand named Ro5-4864, which is known to modulate several mitochondrial signaling pathways. The last three active compounds (LPS 02–25-L-E10, LPS 01–03-L-F03, and LPS 02–13-L-E04) exhibit the same thioamide function. The presence of thioamide function may suggest a mode of action through the chelation of iron. However the pyridazine-3-thiol scaffold was never described for this activity and iron chelators, such as desferrioxamine B, Triapine, or Tachpyridine, are generally much more functionalized in order to efficiently trap atoms of iron. Interestingly, LPS 01-04-L-G10 showed the best activity at 10 *μ*M, with about 60% of pupae after 263 hours and up to 72% after 320 hours. As a drug-like scaffold, the pyridazine-3-thiol is a good candidate for a hit-to-lead program.

## 5. Conclusions

Altogether, the present results open new and promising ways to decipher the molecular basis of frataxin deficiency and to develop original compounds with some efficiency to treat FA. The* Drosophila* based developmental assay, although quite tedious, is robust and should be extremely powerful to further evaluate derivatives of the hits described in this study. In addition it can be completed with other phenotypic assays in flies such as the heart defect rescue assay recently described [31]. These additional developments should help discriminating general or tissue-specific action of the compounds towards development of new drugs for FA.
